# 

**Published:** 1840-04

**Authors:** 


### BOOKS RECEIVED FOR REVIEW.

ENGLISH.

1. Observations on the Management of the Poor in Scotland, and its effect on the health of the great towns. By W. P. Alison, M.D., F.R.S., &c.—Edin. 1840. 8vo, pp. 198. 3s. 6d.

2. The Cyclopædia of Surgery. Parts IV. V. Edited by W. Costello, M.D. (Ankylosis to Burns.) — London, Jan., March, 1840. 5s. each Part.

3. Medical Portrait Gallery. By T. J. Pettigrew, F.R.S. Parts XXIII-V. 3s. each No.

4. A Dictionary of Practical Medicine. By James Copland, M.D., F.R.S. Part VI. (Infection—Insanity.) — London, Jan., 1840. 4s. 6d.

5. The Intimate Structure of Secreting Glands. By John Müller, M.D. With the subsequent discoveries of other authors. By Samuel Solly, F.R.S. With plates.—London, 1839. 8vo, pp. 166. 7s. 6d.

6. Illustrations of Cutaneous Disease. Parts IX. to XV. By R. Willis, M.D., &c.—London, 1839. 5s. each Part.

7. Medical Etiquette; or an Essay upon the laws and regulations which ought to govern the conduct of members of the medical profession in their relation to each other. By Abraham Banks, Esq., Surgeon.—London, 1839. 12mo, pp. 104. 3s. 6d.

8. Rudiments of Animal Physiology. For use in schools and for private instruction. By Dr. G. Hamilton, Falkirk. (Chambers's Educational Course.)—Edin. 1840. 12mo, pp. 104. 1s. 6d.

9. Preservation of the Teeth indispensable to comfort, appearance, health, and longevity. By John Gray, Dentist, M.R.C.S. —London, 1838. 12mo, pp. 77.

10. Practical Observations on various subjects relating to Midwifery. By James Hamilton, M.D., F.R.S.E., Professor of Midwifery in the University of Edinb. Second Edition, revised and enlarged.—Edinb. 1840. 8vo, pp. 418. 12s.

11. Aphorisms on the Treatment and Management of the Insane; with considerations on public and private lunatic asylums, pointing out the errors in the present system. By J. G. Millingen, M.D., &c.—London, 1840. 12mo, pp. 202. 4s. 6d.

12. A Compendium of Materia Medica and Pharmacy, adapted to the London Pharmacopœia. By J. Hunter Lane, M.D., F.L.S., &c.—London, 1840. 12mo, pp. 308. 5s.

13. A Treatise on the Ear; including its Anatomy, Physiology, and Pathology; for which the author obtained a gold medal in the University of Edinburgh. —London, 1840. 8vo, pp. 255. With Plates. 10s. 6d.

14. Animal Mechanism and Physiology; being a plain and familiar exposition of the structure and functions of the human system. Designed for the use of families and schools. By J. H. Griscom, M.D.—New York, 1839. 12mo, pp. 357.

15. A Compendium of Materia Medica, Pharmacy, and Toxicology. For the use of medical students. By D. Spillan, M.D. —London, 1839. Sm. 8vo, pp. 80.

16. Twenty-fifth Annual Report of the Directors of the Glasgow Royal Asylum for Lunatics.—Glasgow, 1839. 8vo, pp. 25.

17. Proceedings of the Border Medical Society. Instituted June 27, 1838.—Kelso, 1838-9. 8vo, pp. 26.

18. Outlines of Physiology; with an Appendix on Phrenology. By P. M. Roget, M.D., F.R.S., &c. Revised with numerous Notes.—Philadelphia, 1839. 8vo, pp. 516.

19. New Remedies; the method of preparing and administering them; their effects on the healthy and diseased economy, &c. By R. Dunglison, M.D., M.A.P.S., Professor of Medicine in the College of Philadelphia, &c.—Philadelphia, 1839. 8vo, pp. 503.

20. A Treatise on the causes and consequences of habitual constipation. By John Burne, M.D., Physician to the Westminster Hospital, &c.—London, 1840. 8vo, pp. 257. 7s. 6d.

21. Elements of the Practice of Medicine. By C. Lendrick, M.D., Queen's Professor of the Practice of Medicine, &c. Part I.—Dublin, 1840. 8vo, pp. 123. 3s.

22. Anatomical, Pathological, and Therapeutic Researches on the Yellow Fever of Gibraltar of 1828. By P. C. A. Louis, M.D. Physician to the Hotel Dieu, &c. Translated from the MS. by G. C. Shattuck, jun. M.D.—Boston, 1839. 8vo, pp. 374.

23. An Atlas of Plates illustrative of the Principles and Practice of Obstetric Medicine and Surgery. By F. H. Ramsbotham, M.D., &c. Nos. I.-III. 8vo, 16 in each. 1s. 6d.

24. Medical Communications of the Massachusetts Medical Society. Vol. II. Parts I.-III.—Boston, 1837-8-9. 8vo, pp. 257.

25. Practical Observations on the Nature and Treatment of Talipes or Clubfoot, particularly of Talipes Varus. By W. M. Coates, M.R.C.S.—London, 1840. 8vo. pp. 42. With Two Plates. 2s.

26. On the Influence of Artificial Light in causing Impaired Vision, and on some Methods of preventing or lessening its injurious action on the Eye. By James Hunter, M.D. Surgeon to the Edinburgh Eye Dispensary.—Edinburgh, 1840. 8vo, pp. 94. With a Plate. 3s. 6d.

27. A Treatise on Syphilis. By Herbert Mayo, F.R.S. Surgeon of Middlesex Hospital.—London, 1840. 8vo, pp. 128. 5s. 6d.

28. On the Anatomy of the Breast. By Sir Astley P. Cooper, Bart., F.R.S., D.C.L., G.C.H., Sergeant-Surgeon to the Queen.—London, 1840. 4to, pp. 193. With a volume of Folio Plates. 3*l*. 3s.

29. Pathological Observations on the Diseases of the Uterus. By Robert Lee, M.D., F.R.S., Physician to the British Lying-in Hospital, &c. Part I.—London, 1840. Folio, pp. 12. With Four Coloured Plates. 14s.

FOREIGN.

1. Untersuchungen zur Physiologie und Pathologie. Von Dr. Fried. Nasse und Dr. Herm. Nasse. Zweiten Bandes, Zweites Heft.—Bonn, 1839. 8vo, pp. 155.

2. Sul Cholera Morbus Avvisi medici di Giuseppe Maria Stilon, M.D. &c.—Malta, 1839. 8vo, pp. xxiii. 61.

3. Die diagnostische bedeutung der einzelnem Symptome der hitzigen Hirnhöhlenwassersucht der Kinder. Von Dr. H. Wolff, prakt. Arzte zu Bonn.—Bonn, 1839. 8vo, pp. 63.

4. Essai Statistique sur la Mortalité du Canton de Genève pendant l'anneé 1838. Par le Dr. Marc d'Espine.—Paris, 1840. 8vo, pp. 130.

5. Mémoires sur les Difformités du Système Osseux. Par le Dr. Jules Guerin, Directeur de l'Institut Orthopédique de la Muette, &c. I. Sur l'Extension sigmoïde et la Flexion dans le traitement des Déviations laterales de l'Epine. II. Sur les Déviations simulées de la Colonne vertébrale. III. Sur le Traitement du Torticolis ancien. IV. Sur l'Etiologie générale des Pieds-bots congénitaux. V. Sur les Variétés anatomiques du Pied-bot congénital. VI. Sur les Caractères généraux du Rachitisme. VII. Vues générales sur l'Etude des Difformités du Système osseux.—Paris, 1838-40. 8vo.

6. Geschichte der neueren Heilkunde. Von Dr. J. F. C. Hecker.—Berlin, 1839. 8vo. pp. 614.

7. Rede zur Feier 45ten Stiftungstages des könig. med.-chir. Friedrich-Wilhelms-Instituts, am 2ten Aug. 1839. Von Dr. J. F. K. Hecker.—Berlin, 1839. 8vo, pp. 20.

### INDEX TO VOL. IX.

OF THE

### BRITISH AND FOREIGN MEDICAL REVIEW.

PAGE

Abdomen, diseases of the . 473
Abortion, criminal, jurisprudence of 36
Acupuncture in neuralgia . 251
Addison & Bright on the Practice of Medicine . . . 461
Air, injections of, in ileus . 221-50
Albers, Dr. on disease of the cæcum 445
Alison, Dr. on the poor in Scotland 544
Amussat, M. on artificial anus . 259
Amputation, Mr. Cooper on . 392
Amputation, spontaneous, in the fœtus 564
Anatomy, text-books on . . 538
Aneurism, popliteal, cured by pressure 277
Aneurism of the aorta . . 548-71
Angioitis, Dr. Sacchero on . 223
Animalcules, parasitic, cause of contagion . . . 400
Animals, microscopical structure of 495
Antagonism, Dr. Henle on . 404
Anus, artificial, new mode of forming 259
Aorta, aneurism of . . 548-71
Apoplexy of the eyes . . 549
Appendix cæci, perforation of . 451
Army, Medical Reports of . 68
Arsenic, use of, in chorea . 271
Arthritis, morphia in . . 252
Ashwell, Dr. on occlusion of the uterus 382
Asphyxia, jurisprudence of . 49
Association, provincial, transactions of 216
Asylums, lunatic, management of 144
Auscultation, researches in . 299
Baer, Von, on the ovum . . 1
Ballingall, Sir G. his military surgery 243
Baly, Dr. his Müller's physiology 244
Barzellotti, Dr. on med. jurisprudence 35
Barry, Dr. on embryology . 1
Baryta in ophthalmia . . 552
Beck, Dr. obituary of . . 295
Bell, Sir C. his discoveries . 96
Belladonna in epilepsy . . 252
Bellingeri, his nervous discoveries 141
Bird, Dr. on charcoal vapours . 377
Bird, Dr. his natural philosophy 529
Blake, Mr. on poisons . . 569
Blane, Sir G. on hydrocephalus . 231
Blood, inoculation with diseased . 245
menstrual, injurious . 545
Bones, analysis of, in mollities . 389
Bonnet, M. on the treatment of varix 252
Books for review . 297-583
Border medical society . . 542
Bougie constructed from fucus . 222
Bouillaud on sanguineous concretions 549
Bowels, diseases of, among soldiers 66
Brain, diseases of, among soldiers 69
mental, physiology of . 222
diseases of the . . 370
tubercles in . . 574
Breast, Sir A. Cooper on . . 538
Brera, Prof. on calculus . . 246
Barnard, Mr. on hydrocephalus . 230
Bright & Addison on the prac. of med. 461
Dr. on spasmodic diseases 431
Burne, Dr. on diseases of the cæcum 445
Bronchitis, acute, treatment of . 488
Cæcum, gastrotomy in disease of 250
tumours of . . 451
inflammation of . . 445
Calculi, chemical relations of . 360
general form of . . ib.
colour, size, &c. 361
magnesian phosphate . 365
phosphate & subphos. of lime ib.
carbonate of lime . 366
oxalate of lime . ib.
lithic acid . . 363
chemical solvents of . 368
xanthic oxide . . 364
dissolution of . . 246
urinary, causes of . 248
Campbell, Mr. on ischuria . 573
Cancer, treatment of, by ligature 260
of the stomach . . 477
Capillaries, structure of . . 518
Carbonated hydrogen, poisoning by 380
Carcinoma uteri, statistics of . 442
Carditis, case of . . 433
Cells, animal, growth of . . 505
Chest, on the diseases of the . 484
Cholera in the British army . 73
Cicatrices in the lungs, new theory of 340
Cities, cause of mortality in . 359
Charcoal vapours, poisoning by . 377
Chorea, use of arsenic in . 271
Club-foot, on the cure of . . 227
College of Surgeons, reform in . 287
Combe, Mr. on phrenology . 190
Concretions, fibrinous, of the heart 386
sanguineous . 549
Condylomata, nature of . . 251

PAGE
Conferva, contagious, animal . 550
Contagion, curious cases of . 571
Contagions, Dr. Henle on . 298
Cooper, Sir A. on dislocation . 391
on the breast . 542
Mr. B. on amputation . 392
hernia . ib.
Copaiba, poisoning from . . 270
Copenhagen, medical topography of 216
Cord, spinal, pathology of . 437
Cornea, nerves of . . 548
Coroners, duties and qualifications of 51
Corpora lutea, true and false . 570
Corpus luteum, structure of . 445
Corrigan, Dr. on opium in rheumatism 274
Coste, M. on embryology . 1
Costello, Mr. his Cyclopædia . 539
Cowpox, identity of, with smallpox 76
origin of . . 78-86
Croup, treatment of . . 573
Crumpling sound a sign of phthisis 313
Cyclopædia of surgery . . 539
Cytoblasts . . . 498
Dead bodies, mode of preserving . 397
Deaths in England, report on . 344
and Wales, in 1837 352
Delirium tremens . . 476
among soldiers . 69
Development of the ovum . 1
Diarrhœa, peculiar form of . 477
Dieffenbach, M. on club-foot . 227
cure of squinting 558
dislocation . 556
Digitalis, its effect on the uterus . 560
Dodd, Mr. on varicose veins . 575
Dubois, M. on medical education 411
Dunglison, Dr. on new remedies 541
Dysentery treated with albumen 552
Education, medical preliminary . 411
Egg, on the incubated . . 396
Eggs, poisoning from . . 267
Elbow, dislocation of . . 556
Elliotson, Dr. his lectures . 461
Embryology, researches in . 1-291
Empyema and pneumothorax . 393
Endosmosis of the egg, Magendie on 290
Entozoon, new . . . 442
Epilepsy, belladonna in . . 252
Ergot, sedative effects of . 563
Eschricht on embryology . 2
Esquirol, M. on lunatic asylums . 144
Expiration, auscultatory characters of 310
Eye, Mackenzie on the . . 543
Eyelids, operation for new . 559
Eyes, apoplexy of . . 549
Fecal tumours . . 447
Farr, Mr. his reports . . 344
new bills of mortality 582
Fever, yellow, among soldiers . 71
malignant, at Pali . 234
observations on . . 467
Fibrine, on softening of . . 436
Fluoric acid in animal substances 396

PAGE
Fournet, M. on auscultation . 299
Fracture, new mode of treating . 271
Gall on phrenology . , 190
Gangrene, dry, case of . . 442
Gastritis . . . 476
Gastrotomy in cæcal disease . 250
Genitals, male, mortification of . 571
Germ vesicle, on the . . 5
Glands, Müller and Solly on the 533
Gout, new treatment of . . 238
Graafian vesicle, contents of . 13
Graves, Dr. his clinical lectures 461
Green, Dr. on cerebral tubercles . 574
Gregory, Dr. his practice of medicine 461
Griscom, Dr. on physiology . 537
Grisolle, M. on cæcal tumours . 445
Gulliver, Mr. on fibrine . . 436
Guy, Dr. on the pulse . . 337-76
Guy's Hospital Reports . . 370
Hemorrhage, constitutional, hereditary 246
Hall, Dr. M.'s case of anasarca . 276
on the nervous system 438
his claims . . 580
Hamilton, Dr. obituary of . 292
on physiology . 534
Hanging, medical jurisprudence of 50
case of . . 264
signs of . . 266
Hannover, Dr. on contagious conferva 550
Hawkins, Mr. on tumour of the neck 440
Heart, diseases of, among troops 70
fibrinous concretions in . 386
gangrene of . . 434
on the action of . . 561
Hearts, natural diversity of . 487
Heat, pungent, sign of pneumonia 489
evolution of, by plants . 545
Henle, Dr. his researches . 398
Hernia cerebri, cases of . 220
Hill, Mr. on lunatic asylums . 144
Hering, M. on cowpox in the cow 76
Hernia, cases of . . 392
Humerus, dislocation of backwards 391
Hydrocephalus, on pressure in . 230
Identity of cowpox and smallpox 76
India, causes of disease in . 564
Indigestion, alkaline . . 273
Infanticide, jurisprudence of . 47
Infants, relative mortality of . 350
Inflammation, diffuse . . 578
Insane, on the treatment of . 144
diet of . . 167
work done by . 170
Insects, Mr. Newport on . 533
Inspiration, physical characters of 308
Intussusception, case of . 221
Irritability, muscular, in paralysis 438
Ischuria renalis, cases of . . 551-73
Italian medicine, remarks on . 226
Jones, Mr. W. on embryology . 291
on the ovum . 2
on the eye . 543
Jurisprudence, medical, Barzellotti on 35

PAGE

Key, Mr. his case of deformed tibia 389

Krauss, Dr. on club-foot . 227

Kreysig, Dr. obituary of . 292

on ischuria . 551

Lactic acid, a gout specific . 239

Lane, Mr. on materia medica . 541

Lead, effects of . . 435

Lee, Dr. on the corpus luteum . 445

Leg, deformity of, cured . 389

Legitimacy, jurisprudence of . 42

Leprosy and yaws . . 531

Lincoln Asylum, account of . 153

Liston, Mr. on anasarca of scrotum 443

Lithotripsy, medicinal . 246

Liver, diseases of, among soldiers 64

abscess, cases of . . 276

on diseases of . . 482

Lizars, Dr. his anatomy . . 538

Lunatics, on the treatment of . 144

Lungs, induration of not indicated by percussion . . 490

Lungs, relative morbidity of the two 334

Mackenzie, Dr. on the eye . 543

M'Divitt, Dr. obituary of . 296

Madder, effect on the bones . 272

Magendie, his discoveries . 134

Malden, Dr. his address . 216

Materia medica, works on . 541

Maxwell, Dr. on yaws . 531

Maygrier, M. his anatomy . 538

Mayo, Mr. his discoveries . 135

Mercurial plasters in smallpox . 249

Mercury, alleged cause of secondary symptoms . . 237

Miasmata, Dr. Henle on . 398

Midwifery, reports on . 220

plates of . . 540

Milk, absorption of . . 248

Mollities ossium, chemistry of . 389

Molluscum, case of . . 221

Monsters, civil rights of . 46

Morphia in arthritis . . 252

Mortality, difference in tables of 347

relative in town and country . 349-57

Mortification of the genitals . 571

Mortality, new bills of . 582

Mouat, Dr. on the causes of Indian diseases . . 564

Mucous membranes, structure of ib.

Müller, M. on the secreting glands 533

Murphy, Dr. on mercury . 237

Muscles, division of, in spine disease 554

Mydriasis, idiopathic . 258

Nails, curvature of . . 548

Nasmyth, Mr. on the teeth . 443

Natural history, importance of . 426

Nerves, structure of . . 517

of the cornea . . 548

Nervous system, discoveries in . 96

diseases of . 492

PAGE

Nervous system, the sensori-volitional 101
the excito-motor ·103
the sympathetic ib.
Dr. Hall on . 580
Neuralgia, acupuncture in . 251
incision in . 258
Newport, Mr. on insects . 533
North of England association . 290
Nosology, statistical, Mr. Farr's . 351
Nunneley, Mr. his anatomy . 538
Oil, cod-fish, in scrofula . 553
Otto, Dr. on Copenhagen . 216
Ophthalmia, baryta in . 552
Belgian . . 557
Orfila on hanging . . 266
Orthopædia, subcutaneous . 227
Osborne, Dr. on thread setons . 578
Ovum, on the development of . 1
Ovum, primary structure of . 507
Paget, Mr. on bone . . 272
Paley, Dr. on mortification . 571
Paterson, Dr. on corpora lutea . 570
Paralysis, irritability in . 438
Pathology and practice, present state of . . 462
Perforation of the stomach . 371
of appendix cæci . 451
Pericardium wanting . . 440
in spasmodic cases . 431
Petrifaction of tissues . 246
Pettigrew, Mr. his portraits . 543
Philosophy, natural, Dr. Bird's . 529
Phrenology, principles of 191-197
progress of . 192
mode of studying . 206
Phthisis, on the first stage of . 299
curability of . . 323
table of signs of . 335
incipient, diagnosis of . 396
Physiology, Müller's elements of 244
treatises on 534-7
Physical sciences, importance of 420
Plague in the army . . 75
Plants, structure of . . . 495
evolution of heat by . 545
Pneumonia, diagnosis of . 489
treatment of . 491
Pneumothorax and empyema . 393
Poisoning, suspected . 270
in England, statistics of 278
by charcoal vapours . 377
by carburetted hydrogen 380
by sulphuric acid . 391
Poisons, mode of action of . 569
Portrait Gallery, medical . 543
Poor, Dr. Alison on . . 544
Parker, Mr. on syphilis . 239
Practice of physic, treatises on . 461
Prinz, on regeneration of the vaccine 76
Prostate, disease of . . 260
Pulse in phthisis, character of 337-396

| | PAGE |
|---|---|
| Purkinje on the ovum . . | 5 |
| Ramsbotham, Dr. his obstetric tables | 540 |
| Ranken, Dr. on malignant fever . | 234 |
| Reed, Dr. on practice of physic . | 461 |
| Reform, medical . . | 281 |
| Registrar-General, his report . | 344 |
| Remak, Dr. on menstrual blood . | 545 |
| Remedies, new, Dr. Dunglison on | 541 |
| Respiration, normal and anormal . | 304 |
| state of, in phthisis . | 329 |
| Restraint of the insane considered | 154 |
| Retrovaccination . . | 88 |
| Revaccination in Denmark . | 292 |
| Rheumatism, opium in . . | 274 |
| pulse in . . | 485 |
| Roget, Dr. on physiology . | 534 |
| Ronchi in phthisis . . | 329 |
| Rutter, Dr. obituary of . . | 295 |
| Ryan, Dr. his obstetric tables . | 540 |
| Sacchero, Dr. his clinical report . | 223 |
| Schwann, on the structure of animals | 495 |
| Schleiden on the structure of plants | ib. |
| Shaw, Mr. on Sir C. Bell's discoveries | 96 |
| Smallpox, identity of, with cowpox | 76 |
| mercurial plasters in . | 249 |
| mortality from . | 353 |
| Scharling, M. on calculi . | 360 |
| Science, general, its value . | 428 |
| Scrofula, cod-oil in . . | 553 |
| Setons, on thread . . | 578 |
| Solly, Mr. on the glands . . | 533 |
| Sounds, new pulmonary . . | 315-19 |
| Spasmodic diseases, pericardial . | 431 |
| Spinal cord, diseases of . . | 493 |
| Spine, on curvature of . . | 444 |
| cured, treated by incision | 554 |
| Spontaneous amputation in the fœtus | 564 |
| Spurzheim on phrenology . | 190 |
| Stokes, Dr. his lectures . . | 461 |
| Stomach, diseases of . . | 66 |
| perforation of . . | 371 |
| Strabismus cured by operation . | 558 |
| Structure of plants and animals . | 495 |
| Succession, rights of . . | 44 |
| Sulphuric acid, poisoning from . | 391 |
| Superfœtation, jurisprudence of . | 42 |
| Surgery, military . . | 243 |
| Sweatman, Dr. obituary of . | 296 |
| Sigmond, Dr. on tea . . | 241 |
| Sympathies, nervous . . | 404 |
| Synovial tumours, new treatment of | 554 |
| Syphilis, on the treatment of . | 239 |
| Tabes, mesenterica . . | 477 |
| Taylor, Mr. on perforation . | 371 |
| Tea, its effects . . | 241 |
| Teeth, on the capsules of . | 443 |
| microscopical structure of | 513 |
| Tetanus and trismus infantum . | 531 |
| Thiele, Dr. on cowpox and smallpox | 76 |
| Thomson, Dr. on indigestion . | 273 |
| Tissues, microscopical varieties of | 509 |
| Todd, Dr. on the mucous membrane | 565 |
| Tongue, malignant disease of . | 433 |
| Towne, Mr. on the egg . . | 396 |
| Toxicology, Barzellotti on . | 51 |
| Transactions of Provincial Association . . | 216 |
| medico-chirurgical | 431 |
| Tumour of the neck, peculiar . | 440 |
| Tumours, synovial, new treatment of | 554 |
| Turin, clinical medicine at . | 223 |
| Typhlo-enteritis . . . | 445 |
| Ulcers, malignant, treatment of . | 553 |
| Urine, vomiting of . , | 551 |
| Uterus, expulsion of . . | 261 |
| occlusion of . . | 263-381 |
| cancer, statistics of . | 442 |
| influence of digitalis on . | 560 |
| Uvula, peculiar affection of . | 219 |
| Vaccination, present state of . | 76 |
| Vaccine lymph, regeneration of . | ib. |
| Vagina, sloughing of . . | 262 |
| Valentin, Dr. on the ovum . | 1 |
| Varicocele, case of . . | 390 |
| Varix of the legs, treatment of . | 252 |
| Veins, varicose, treatment of . | 575 |
| Voice, state of, in phthisis . | 329 |
| Walker, Mr. A. his discoveries . | 98 |
| Walter, Dr. Von, on generation . | 1 |
| West Indies, sickness in . | 63 |
| Wilson, Dr. on the croup . | 573 |
| Wounds, jurisprudence of . | 61 |
| Yaws and leprosy, Dr. Maxwell on | 531 |
| Yeast-plant, account of . . | 579 |

END OF VOL. IX.

PRINTED BY C. ADLARD, BARTHOLOMEW CLOSE.

